# Recycling of CO_2_ by Hydrogenation of Carbonate Derivatives to Methanol: Tuning Copper–Oxide Promotion Effects in Supported Catalysts

**DOI:** 10.1002/cssc.202000166

**Published:** 2020-03-13

**Authors:** Jonglack Kim, Norbert Pfänder, Gonzalo Prieto

**Affiliations:** ^1^ Heterogeneous Catalysis Max-Planck-Institut für Kohlenforschung Kaiser-Wilhelm-Platz 1 45470 Mülheim an der Ruhr Germany; ^2^ Max-Planck-Institut für Chemische Energiekonversion Stiftstrasse 34–36 45470 Mülheim an der Ruhr Germany

**Keywords:** carbon dioxide fixation, copper, hydrogenation, nanoparticles, supported catalysts

## Abstract

The selective hydrogenation of organic carbonates to methanol is a relevant transformation to realize flexible processes for the recycling of waste CO_2_ with renewable H_2_ mediated by condensed carbon dioxide surrogates. Oxide‐supported copper nanoparticles are promising solid catalysts for this selective hydrogenation. However, essential for their optimization is to rationalize the prominent impact of the oxide support on performance. Herein, the role of Lewis acid centers, exposed on the oxide support at the periphery of the Cu nanoparticles, was systematically assessed. For the hydrogenation of propylene carbonate, as a model cyclic carbonate, the conversion rate, the apparent activation energy, and the selectivity to methanol correlate with the Lewis acidity of the coordinatively unsaturated cationic sites exposed on the oxide support. Lewis sites of markedly low and high electron‐withdrawing character promote unselective decarbonylation and decarboxylation reaction pathways, respectively. Supports exposing Lewis sites of intermediate acidity maximize the selectivity to methanol while inhibiting acid‐catalyzed secondary reactions of the propanediol product, and thus enable its recovery in cyclic processes of CO_2_ hydrogenation mediated by condensed carbonate derivatives. These findings help rationalize metal–support promotion effects that determine the performance of supported metal nanoparticles in this and other selective hydrogenation reactions of significance in the context of sustainable chemistry.

## Graphical abstract

**A pillar of support**: The Lewis acidity of coordinatively unsaturated centers (*cus*) on the oxide support tunes promotion effects in oxide‐supported copper nanoparticles as catalysts for selective hydrogenation of propylene carbonate. Intermediate Lewis acidity of the oxide maximizes the selectivity to methanol while preserving the propanediol molecular backbone, as required for cyclic processes of CO_2_ recycling to methanol via carbonate intermediates.

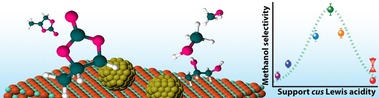

## Introduction

In supported metal catalysts, the role of the oxide support often goes beyond that of a high‐surface‐area carrier that increases the metal dispersion and mechanical stability. Oxide species are known to exert promotion effects that profoundly modify the catalytic performance and stability of the metal species with which they are in contact.^1^ Metal/oxide promotion effects are particularly significant for reactions that require the activation of polarized bonds (C−O, N−O, S−O, etc.) on “oxophilic” oxide surfaces to operate in conjunction with classical metal‐catalyzed elementary steps, for example, H_2_ dissociation.^2^ Identifying experimentally accessible physicochemical descriptors as a means to reach a quantitative, ideally predictive, description of such metal/oxide promotion effects is currently a major goal. Such insights are expected to set a firmer basis for a deeper understanding of existing catalyst systems and a more rational development of innovative catalysts and processes.

Catalysis by nanoparticles of coinage metals, that is, Au, Ag, and Cu, is particularly dominated by metal/oxide promotion effects. This is in part associated with the relatively low oxophilicity and remarkably low carbophilicity of these metals, which often require the concerted action of oxide species to activate reactants with polar bonds. For example, copper/oxide interfaces have been proposed to play a pivotal role in the very rich, but also strongly support‐dependent, reactivity of copper nanoparticles dispersed on oxide carriers. This includes reactions of particular significance for a more sustainable chemical industry, such as selective hydrogenation of (biogenic) oxygenated compounds,^3^ alcohol synthesis from syngas^4^ and CO_2_,^5^ lignin depolymerization,^6^ and (bio)ester hydrogenolysis.^7^

The hydrogenation reactions of organic derivatives of CO_2_, that is, carbonates, carbamates, and ureas to methanol are of significant interest in the context of chemical CO_2_ recycling, and are archetypal catalytic reactions in which the activation of highly polarized bonds is central to the overall selectivity. In certain scenarios, the hydrogenation of CO_2_ to methanol with H_2_ of renewable origin is considered a feasible route to chemically recycle waste CO_2_ streams into versatile platform chemicals and to store renewable energy in a liquid vector.^8^ By surmounting some of the limitations envisaged for direct CO_2_ hydrogenation processes, the aforementioned organic derivatives could play a substantial role as high‐density, and therefore cost‐effectively transportable, intermediate CO_2_ couriers and thus facilitate the bridging of remote point sources of waste CO_2_ and renewable H_2_, as well as buffering the transient fluctuations that are inherent to both supplies (Scheme S1 in the Supporting Information).^9^ These compounds can be obtained with high selectivity by reaction of CO_2_ with different organic compounds, such as alcohols, glycols, epoxides, and amines.^10^ In a separate catalytic step, organic CO_2_ derivatives can be hydrogenatively converted in the presence of H_2_ as coreactant. If the latter transformation is achieved with high selectivity, the CO_2_‐derived carbon may act as the precursor for methanol in a reaction that, unlike direct CO_2_ hydrogenation, is not bound by thermodynamic limitations to the methanol yield, while the organic residues can be recovered and recycled for further CO_2_ capture, and a cyclic overall process consisting of a net conversion of CO_2_ and H_2_ to methanol is enabled.

Molecular catalysts based on Ru and Ir have been designed to achieve high reaction rates and product selectivities for the selective hydrogenation of organic CO_2_ derivatives in solution.^11^ However, solid catalysts are highly preferred because they enable effective product recovery and catalyst recycling. Catalysts based on supported copper nanoparticles have been proposed for the heterogeneously catalyzed hydrogenation of organic carbonates to methanol.^12^ Stark differences in activity and selectivity have been reported as a function of the nature of the oxide support. However, in spite of the significance of these oxide‐support effects for the rational development of advanced catalysts, they remain poorly understood at present.

Herein, we address the promotion effects of peripheral Lewis oxide centers on the Cu‐catalyzed hydrogenation of propylene carbonate, as a model organic carbonate, to methanol. For a series of model Cu catalysts, the surface Lewis acidic (electron‐accepting) character of the peripheral oxide support was systematically modified and quantitatively assessed in a broad study space. This physicochemical feature is shown to be a rather general and quantitative descriptor for both activity and selectivity, and thus a suitable design parameter for optimized copper‐based catalysts.

## Results and Discussion

### Characterization of oxide support materials

**Figure 1:**
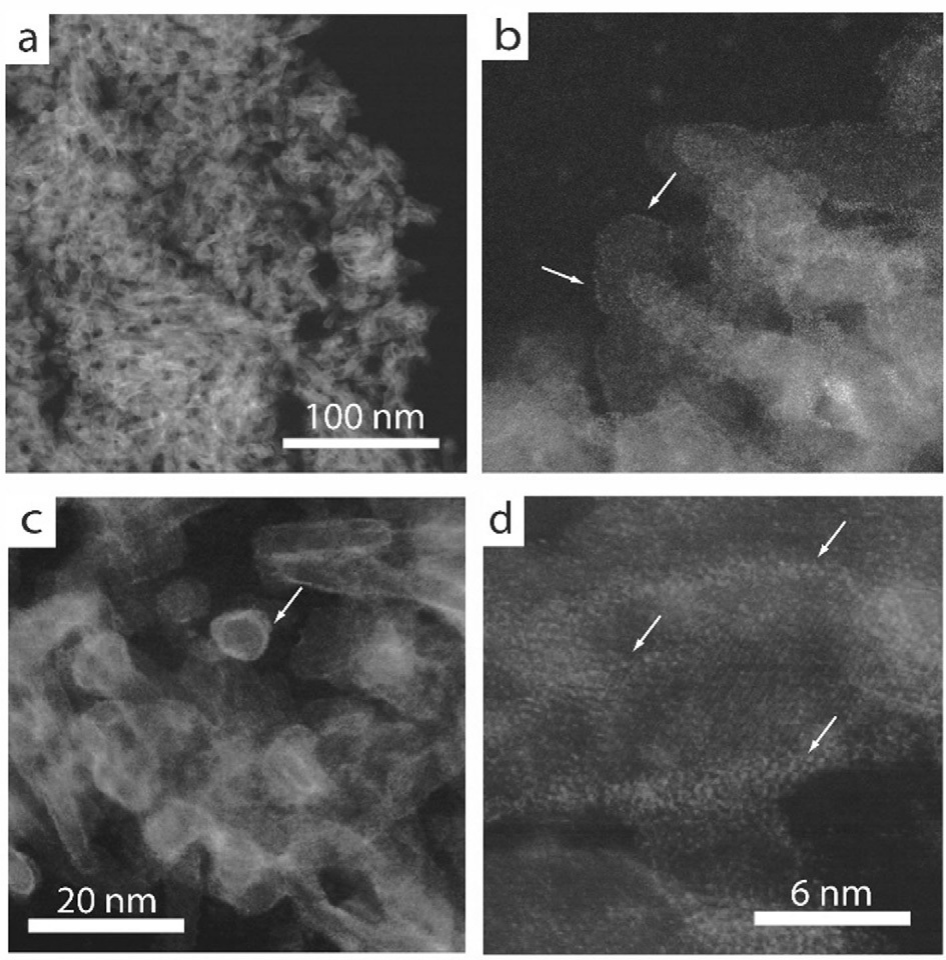
Representative C_s_‐HAADF‐STEM images of (a, b) TaO_*x*_@Al_2_O_3_ and (c, d) SmO_*x*_@Al_2_O_3_ supports consisting of oxide overlays on a high‐surface‐area γ‐Al_2_O_3_ substrate. Arrows point to the higher‐*Z*‐contrast oxide overlay observed as a rim around the γ‐Al_2_O_3_ nanocrystals. In d), samarium atoms in the SmO_*x*_ overlay are resolved.

To synthesize a series of oxide support materials showing similar textural properties (specific surface area, pore volume, and diameter) but different surface Lewis acidities in a broad study space, the surface of a mesoporous γ‐Al_2_O_3_ support was overlaid with different transition‐metal and lanthanide oxides (MO_*x*_; M=Sm, Y, Sc, Zr, Ta) of markedly different intrinsic Lewis acidity. In all cases, the surface coverage of M atoms (M_at_) was set to approximately 4.5–5.0 M_at_ nm^−2^, which has been previously identified to correspond to the monolayer content of different oxides on γ‐Al_2_O_3_.5c, 13 The resulting series of support oxides is hereafter denoted MO_*x*_@Al_2_O_3_, in which MO_*x*_ is the overlay oxide. XRD did not detect crystallites of the MO_*x*_ species (Figure S1 in the Supporting Information). All materials, including the neat γ‐Al_2_O_3_ substrate, showed type IV N_2_ physisorption isotherms characteristic of mesoporous materials (Figure S2 in the Supporting Information), with essentially identical Al_2_O_3_‐normalized specific surface areas (277±21 m^2^ g^−1^) and mesopore volumes (0.70±0.05 cm^3^ g^−1^), as listed in Table 1. These results prove the absence of any significant mesopore plugging following the deposition of MO_*x*_, which would have blocked part of the porosity of the alumina carrier to N_2_ uptake. In addition, the MO_*x*_@Al_2_O_3_ oxides showed an average mesopore size of 9.2±0.6 nm, that is, approximately 2 nm narrower than that of the pristine γ‐Al_2_O_3_ carrier. This reduction in mesopore size agrees reasonably well with that expected on deposition of a monolayer of MO_*x*_ on the inner wall of the Al_2_O_3_ mesopores. It is therefore inferred that the MO_*x*_ species are in all cases deposited as an amorphous, essentially 2 D overlay on the Al_2_O_3_ surface. Indeed, spherical aberration (C_s_)‐corrected high‐angle annular dark‐field scanning‐transmission electron microscopy (HAADF‐STEM) could provide direct visual proof for the existence of a (few‐)atom‐thick oxide overlay enveloping the γ‐Al_2_O_3_ nanocrystals for selected materials, for example, TaO_*x*_@Al_2_O_3_ and SmO_*x*_@Al_2_O_3_, for which the *Z* contrast of the M atoms (M=Ta, Sm) relative to the Al_2_O_3_ matrix was the highest among the series of materials (Figure 1).

**Table 1:** Chemical composition and textural and surface electronic properties of MO_*x*_@Al_2_O_3_ oxide supports and metal dispersion in the corresponding copper‐based catalysts.

Material	Composition		Textural properties^[a]^			*cus* Lewis acidity^[b]^	*d*(Cu)^[i]^ [nm]
	M^[c]^ [wt %]	*δ* ^[d]^ [at nm^−2^]	*S*_BET_^[e]^ [m^2^ $g_{Al_{2}O_{3}}^{-1}$ ]	*V*_p_^[f]^ [cm^3^ $g_{Al_{2}O_{3}}^{-1}$ ]	*d*_p_^[g]^ [nm]	*η* ^[h]^ [eV]	
Al_2_O_3_	–	–	265	0.80	11.5	2.50	6.7
ScO_*x*_@Al_2_O_3_	9.4	4.5	255	0.65	9.4	2.45	4.6
SmO_*x*_@Al_2_O_3_	23.8	4.9	265	0.71	9.6	2.38	16.0
YO_*x*_@Al_2_O_3_	14.5	4.6	270	0.78	9.9	2.40	10.5
ZrO_*x*_@Al_2_O_3_	15.4	4.7	285	0.67	8.6	2.48	8.9
TaO_*x*_@Al_2_O_3_	24.1	4.5	310	0.71	8.4	2.56	4.3

[a] As determined by N_2_ physisorption. [b] Relative Lewis acidity of surface‐exposed *cus*, determined by UV/Vis spectroscopy with 1,2‐dihydroxyanthraquinone as surface probe molecule. [c] Metal loading corresponding to the MO_*x*_ overlay oxide. [d] Surface‐specific M loading. [e] Brunauer–Emmett–Teller (BET) specific surface area, normalized per unit mass of Al_2_O_3_. [f] Total pore volume, normalized per unit mass of Al_2_O_3_. [g] Barrett–Joyner–Halenda (BJH) average pore diameter. [h] Peak energy for the lowest‐energy IMCT of adsorbed 1,2‐dihydroxyanthraquinone as determined by UV/Vis spectroscopy after surface saturation with the probe molecule at room temperature. The experimental error for this parameter was determined to be <0.01 eV from independent repetitions. [i] Average copper nanoparticle size, as determined by XPS after catalyst reduction for Cu/MO_*x*_@Al_2_O_3_ with a copper content of 1.5 Cu_at_ nm^−2^.

**Figure 2:**
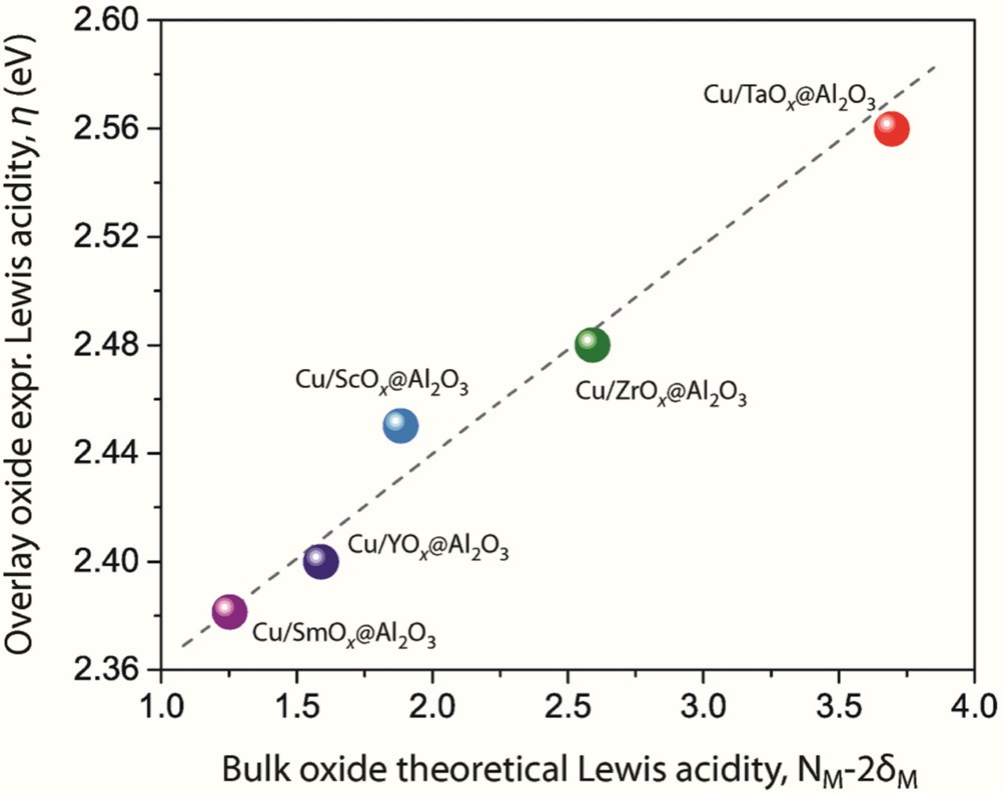
Comparison between the surface acidity determined experimentally for the overlay MO_*x*_ oxides supported on γ‐Al_2_O_3_ with the theoretical (Lewis) acidity of the corresponding bulk oxides, as defined by Jeong et al.,^14^ that is, represented by *N*_M_−2 *δ*_M_, in which *N*_M_ is the formal oxidation state and *δ*_M_ the Sanderson partial charge of the cations in the bulk oxides: Sm_2_O_3_, Y_2_O_3_, Sc_2_O_3_, ZrO_2_, and Ta_2_O_5_.

Notable uniformity in textural properties was achieved in the series of MO_*x*_@Al_2_O_3_ support materials, which would otherwise not be possible should bulk oxides be employed. Moreover, the incorporation of the different oxide overlays on the common Al_2_O_3_ substrate resulted in a series of materials spanning a broad range of surface Lewis acidity. Lewis acidity in oxides stems from coordinatively unsaturated metal sites (*cus*) exposed on their outer surface. The relative Lewis acidity of the *cus* on the surface of the oxide support materials was quantified by means of UV/Vis spectroscopy with 1,2dihydroxyanthraquinone (alizarin) as a surface probe molecule, as described elsewhere.^14^ The lowest intramolecular charge transfer (IMCT) from the catechol subunit to the polycyclic system of the probe molecule appears as a band around 505 nm, in the visible range of the spectra (Figure S3 in the Supporting Information). This energy is hereafter denoted the spectroscopic parameter *η*, and it is plotted in Figure 2 versus the theoretical Lewis acidity of the corresponding bulk‐type MO_*x*_ oxide for the entire series of MO_*x*_@Al_2_O_3_ materials.^14^ The linear correlation observed demonstrates that the ranking of Lewis acidities established experimentally for the *cus* exposed on the surface of the Al_2_O_3_‐supported oxide overlays matches well with that expected for the corresponding bulk‐type oxides, and a broad range of acidity is covered.

### Characterization of copper‐based catalysts

Copper was incorporated in the series of MO_*x*_@Al_2_O_3_ support materials by incipient wetness impregnation. The nominal surface copper content was generally set to 1.5 Cu_at_ nm^−2^. On selected supports, catalysts with higher surface Cu contents of 3.0 and 4.5 Cu_at_ nm^−2^ were also synthesized. The thermal treatment following impregnation was designed to ensure full dehydration of the copper nitrate precursor at mild temperature (343 K) in a flow of N_2_, prior to its decomposition at higher temperatures. Previous studies have shown that this route leads to highly dispersed Cu^II^ species on the surface of the oxide support.^15^ XRD of the as‐calcined catalysts showed no signs of CuO crystals for catalysts with a Cu content of 1.5 Cu_at_ nm^−2^ (2.5–4 wt %), indicative of the very high degree of copper dispersion achieved (Figure S4 in the Supporting Information). Hydrogen temperature‐programmed reduction (H_2_‐TPR) experiments showed H_2_ consumptions, corresponding to the reduction of Cu^II^ species to their metallic state, peaking in the temperature range of 425–510 K. On the basis of this reducibility study, a treatment at 523 K under flow of 20 %H_2_/N_2_ was selected to activate the catalysts prior to catalysis (Figure S5 in the Supporting Information).

Following in situ catalyst reduction, X‐ray photoemission spectroscopy (XPS) showed Cu 2p_3/2_ binding energies (BEs) of 932.2±0.4 eV for the entire series of materials (Table S1 in the Supporting Information), with Cu L_3_M_4,5_M_4,5_ at a kinetic energy of approximately 919 eV. Jointly, these observations point to full reduction of copper species to the metallic state, in line with what could be inferred from the H_2_‐TPR profiles. The overlay MO_*x*_ oxides, however, showed no appreciable reduction following the H_2_ activation step at 523 K (Table S1 in the Supporting Information). Only in the case of TaO_*x*_ could a contribution from a sub‐oxide species be detected. However, this contribution was determined to account for less than 15 % of the tantalum species on the catalyst surface. These results testify to the low reducibility of the oxides deposited as an overlay on the surface of the γ‐Al_2_O_3_ support.

**Figure 3:**
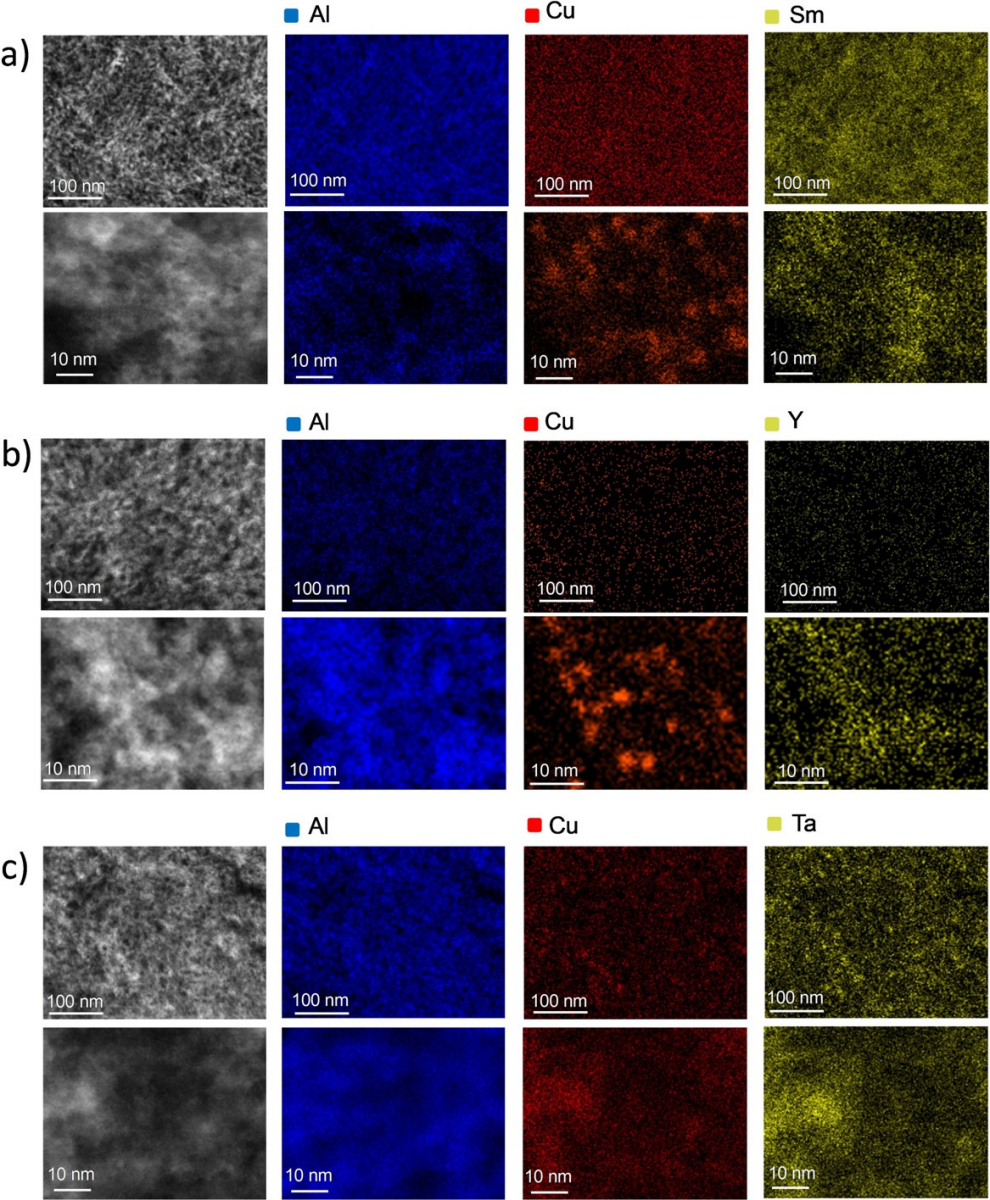
Representative C_s_ HAADF‐STEM images (left) and the corresponding EDX compositional maps for nanometer‐thick cross sections of a) Cu/SmO_*x*_@Al_2_O_3_, b) Cu/YO_*x*_@Al_2_O_3_, and c) Cu/TaO_*x*_@Al_2_O_3_ catalysts, with a copper content of 1.5 Cu_at_ nm^−2^ after reduction.

C_s_‐corrected HAADF‐STEM was coupled with energy dispersive X‐ray (EDX) spectroscopy to investigate the dispersion of Cu and MO_*x*_ species on the surface of the reduced catalysts. Figure 3 shows representative STEM images and compositional EDX mappings, collected at both the mesoscopic and nanometric length scales, respectively, for selected catalysts after reduction. A remarkably uniform mesospatial distribution of the MO_*x*_ overlay oxides could be ascertained in all cases, without any appreciable zoning or agglomeration. Owing to the relatively high *Z* contrast contributed by the MO_*x*_@Al_2_O_3_ support oxides, the presence of Cu nanoparticles (with sizes of ≈6–15 nm) could only be ascertained in few catalysts, with the assistance of EDX compositional maps to identify copperenriched nanoscale regions. For other materials, however, direct visualization of the Cu nanoparticles proved challenging owing to limited *Z*‐contrast differences and smaller Cu nanoparticle sizes (Figure 3 c and Figure S6 in the Supporting Information).

Given the limitations associated with gas chemisorption methods to reliably determine Cu dispersion for a series of catalysts supported on a wide range of oxides,^16^ we resorted to quantitative analysis of the XP spectra of the in‐situ‐reduced catalysts as a means to evaluate Cu dispersion and average particle size in the series of Cu/MO_*x*_@Al_2_O_3_ catalysts. This bulk‐sampling method provides a quantitative assessment that is complementary to the local electron‐microscopy analysis. As summarized in Table 1 and Figure S7 in the Supporting Information, the average Cu nanoparticle sizes determined for the series of catalysts synthesized with a copper content of 1.5 Cu_at_ nm^−2^ were in the range of 4–16 nm. At this constant Cu surface content, the average Cu nanoparticle size increased for catalysts supported on increasingly Lewis basic oxides, notably YO_*x*_@Al_2_O_3_ and SmO_*x*_@Al_2_O_3_. This trend may be associated with partial hydrolysis of the copper nitrate precursor during impregnation and drying, which is known to result in a greater extent of metal agglomeration15c and is expected to be facilitated on oxide supports of basic character by local pH increases of the mesoporeconfined impregnation solution during catalyst synthesis. Catalysts with similar average Cu nanoparticle sizes could also be synthesized on the most Lewis acidic TaO_*x*_@Al_2_O_3_ support by increasing the surface‐specific Cu loading (Figure S7 in the Supporting Information).

### Hydrogenation of propylene carbonate

The catalytic performance of the set of Cu/MO_*x*_@Al_2_O_3_ model catalysts was evaluated in the hydrogenation of propylene carbonate in the liquid phase. Methanol, propane(di)ols, CO, and CO_2_ were the major reaction products. Light hydrocarbons (C_1_–C_3_) were detected under certain reaction conditions, albeit only in small amounts (<5 %). Under identical reaction conditions, blank experiments with the Cu‐free MO_*x*_@Al_2_O_3_ oxide support materials showed insignificant propylene carbonate conversion (<5 % after 24 h), that is, the presence of Cu is essential for catalysis.

**Figure 4:**
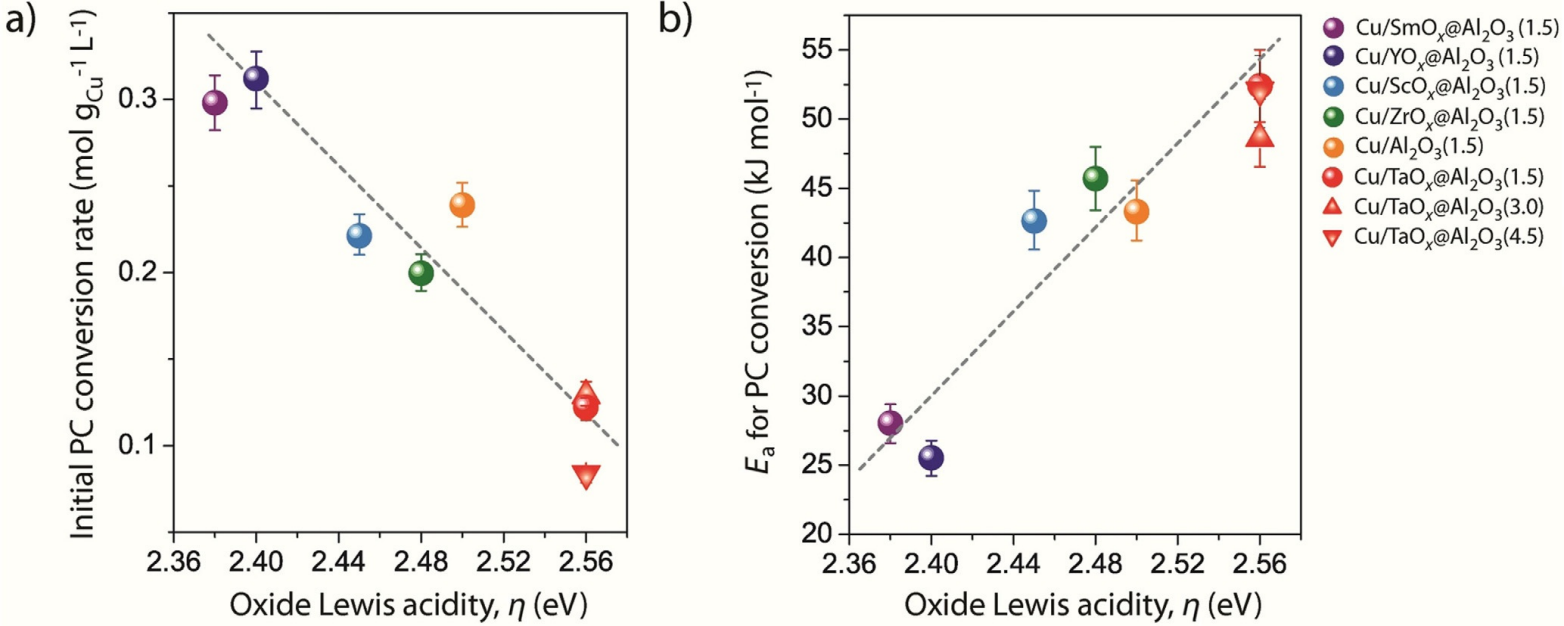
Impact of the Lewis acidity of *cus* exposed on the surface of the oxide support on the catalytic activity. Evolution of a) the initial propylene carbonate (PC) conversion rate; and b) the apparent activation energy with the spectroscopic parameter *η* in the hydrogenation of propylene carbonate for the series of Cu/MO_*x*_@Al_2_O_3_ catalysts. Reaction conditions: *T*=453 K, *p*(H_2_)=40 bar, initial propylene carbonate concentration=0.25 m in 1,4‐dioxane, catalyst concentration=0.15 mm Cu. Error bars represent the standard error from three independent tests with selected catalysts. Dashed lines are included as guides to the eye.

Remarkably, as shown in Figure 4 a, the initial propylene carbonate conversion rate showed strong dependence on the Lewis acidity of the *cus* on the oxide support. The highest reaction rates (0.29–0.31 mol g_Cu_^−1^ L^−1^ min^−1^) were registered for Cu nanoparticles deposited on oxide supports exposing surface *cus* of the lowest Lewis acidity, that is, SmO_*x*_@Al_2_O_3_ and YO_*x*_@Al_2_O_3_. With progressively increasing surface Lewis acidity of the support, the reaction rate decreased progressively by a factor of approximately three over the entire study space.

To assess whether a secondary reaction pathway involving hydrogenation of CO_2_ contributed to the observed methanol formation rates, a set of control experiments was conducted with CO_2_ and H_2_ as reactants. In these tests, the CO_2_ partial pressure in the gas feed was set to 10 bar, that is, simulating the case in which propylene carbonate had initially been fully decarboxylated under the reaction conditions. However, the methanol formation rates registered from CO_2_ were approximately one to two orders of magnitude lower than those observed from propylene carbonate (see Table S2 and the accompanying discussion in the Supporting Information). These results confirmed the prevalence of a direct carbonatehydrogenation pathway as the major methanol production route.

The differences observed in the overall carbonate conversion rates among the series of catalysts might arise from active sites of different intrinsic reactivity and/or differences in the relative abundance of identical active sites. However, an analysis of the initial carbonate conversion rates in the temperature range of 413–473 K (Figure S8 in the Supporting Information) revealed a stark dependence of the apparent activation energy *E*_a_ for carbonate conversion on the oxide *cus* Lewis acidity. As shown in Figure 4 b, *E*_a_ increased rather linearly with the spectroscopic parameter *η*, from 28 kJ mol^−1^ for Cu/SmO_*x*_@Al_2_O_3_ to 53 kJ mol^−1^ for Cu/TaO_*x*_@Al_2_O_3_. It is hence inferred that the differences in reactivity are indeed connected to reaction pathways with different overall energetic barriers operating on different catalysts, rather than simply to differences in the surface density of active sites.

**Figure 5:**
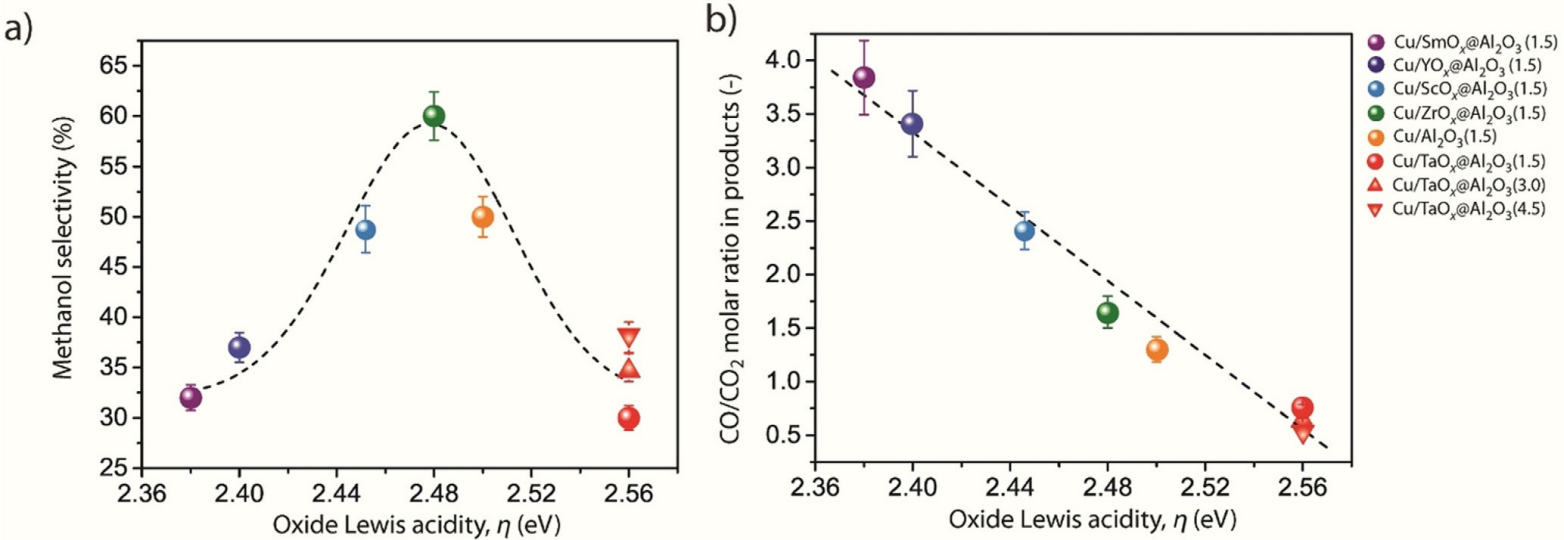
Impact of the Lewis acidity of *cus* exposed on the surface of the oxide support on the reaction selectivity. Evolution of a) the selectivity to methanol and b) the CO/CO_2_ molar ratio in the products with the spectroscopic parameter *η* in the hydrogenation of propylene carbonate with the series of Cu/MO_*x*_@Al_2_O_3_ catalysts. Reaction conditions: *T*=453 K, *p*(H_2_)=40 bar, initial propylene carbonate concentration=0.25 m in 1,4‐dioxane, catalyst concentration=0.15 mm Cu, propylene carbonate conversion: 80 %. Error bars represent the standard error from three independent tests with selected catalysts. Dashed lines are included as guides to the eye.

To assess the influence of the support Lewis acidity on product selectivity, the reaction product pattern was examined as a function of carbonate conversion for all catalysts at a reaction temperature of 453 K. Relatively steady product selectivity patterns were obtained in all cases after a propylene carbonate conversion of 70 % had been reached (Figure S9 in the Supporting Information). Figure 5 a depicts the evolution of the methanol selectivity, at a constant propylene carbonate conversion of 80 %, as a function of *η*. A volcano dependence is observed, according to which the selectivity to methanol is maximized (>60 %) for Cu nanoparticles deposited on oxides exposing *cus* of intermediate Lewis acidity, namely ZrO_*x*_@Al_2_O_3_, and decreases remarkably as the *cus* sites exposed on the surface of the support become either more electron‐donating or more electron‐accepting.

The copper particle size is another parameter that may potentially affect the catalytic performance. Structure‐sensitivity phenomena, that is, particle‐size‐dependent turnover frequencies, have been reported for hydrogenation reactions with supported copper nanoparticles in the sub‐10 nm size regime.^17^ However, analysis of the catalytic performance for catalysts with different average Cu nanoparticle sizes in the range of 4.3–15.2 nm supported on a common oxide carrier, that is, TaO_*x*_@Al_2_O_3_, showed very similar apparent activation energies (Figure 4 a) and product selectivities at a constant carbonate conversion of 80 % (Figure 5). These results further confirm that the Lewis acidity of the oxide support is a dominant factor for reactivity. In addition, these findings suggest that *cus* on the surface of the oxide species, at the periphery of the copper nanoparticles, are involved in a kinetically relevant reaction step.

**Figure 6:**
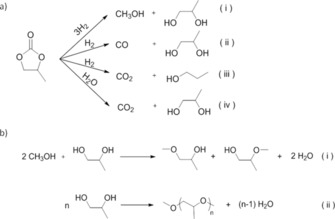
a) Schematic representation of the possible reaction pathways for the conversion of propylene carbonate under the applied reaction conditions: (i) desired selective hydrogenation to methanol and 1,2‐propanediol; (ii) direct decarbonylation pathway; (iii) direct decarboxylation pathway; and (iv) hydrolysis to CO_2_ and 1,2‐propanediol. b) Illustrative acid‐catalyzed (i) esterification with methanol and (ii) oligomerization secondary reactions for the 1,2‐propanediol primary product.

Not only the overall selectivity to carbon oxides but also the composition of these gas‐phase byproducts depended markedly on the Lewis acidic character of the peripheral *cus*. As shown in Figure 5 b the CO/CO_2_ molar ratio in the products at a carbonate conversion of 80 % decreased linearly with increasing *η*. Different side‐reaction pathways might be responsible for the production of CO and CO_2_ under the reaction conditions applied for propylene carbonate hydrogenation. Figure 6 a summarizes these possible reaction pathways (ii)–(iv), together with the desired selective hydrogenation to methanol and propanediol [pathway (i)]. Carbon monoxide formation might be the result of decarbonylation of the carbonate substrate [pathway (ii)]. It is therefore inferred from all these catalytic results that oxide supports exposing surface *cus* with increasingly stronger Lewis basic character promote fast propylene carbonate decarbonylation. This reaction pathway is by and large responsible for both the higher carbonate conversion rates and the higher selectivity to CO observed as the spectroscopic parameter *η* decreases (Figures 4 and 5, respectively).

**Figure 7:**
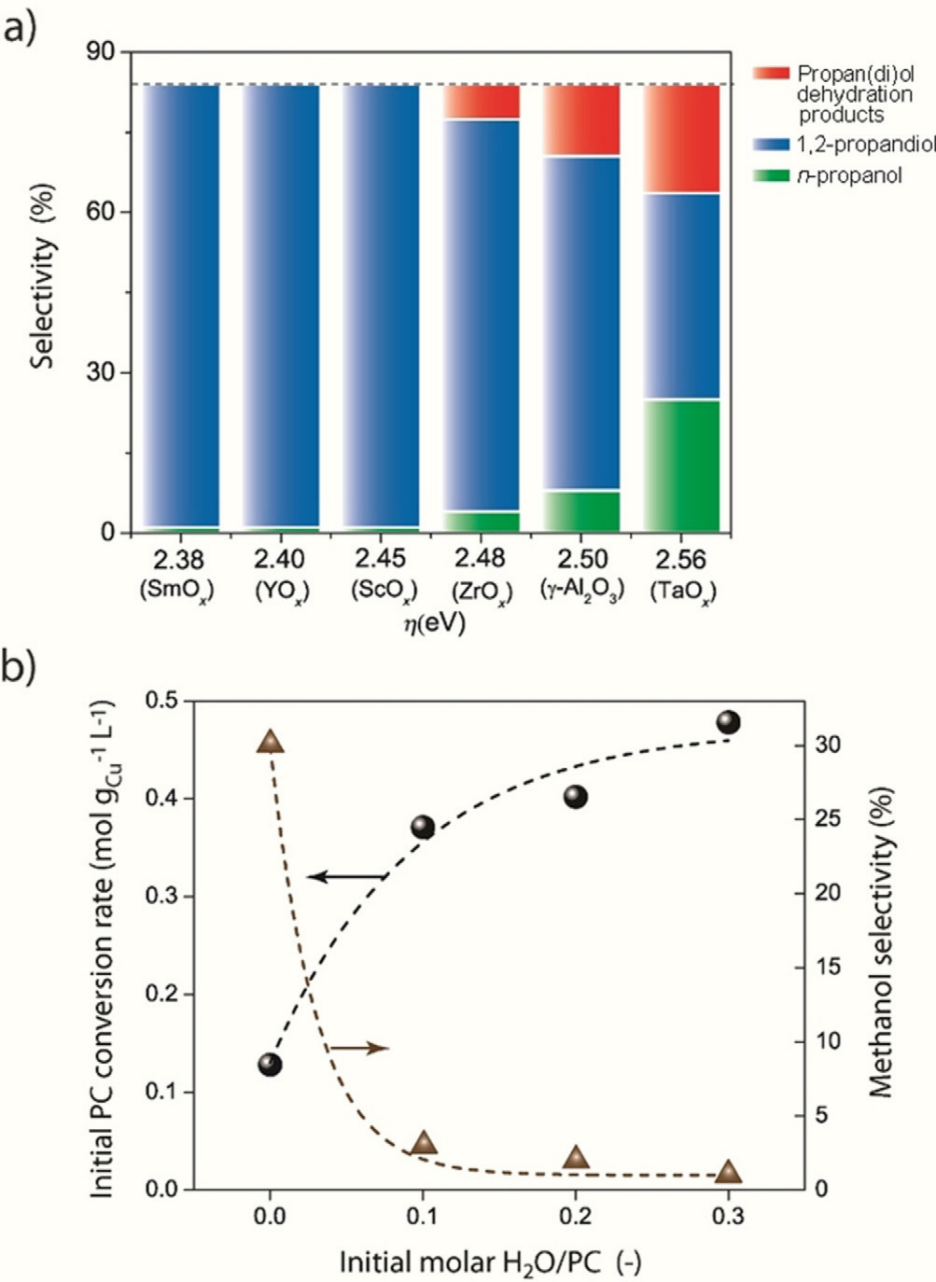
Contribution of dehydration secondary reactions and water byproducts to the catalytic performance. a) Evolution of the selectivity to *n*‐propanol, 1,2‐propanediol, and soluble products of 1,2‐propanediol dehydration reactions as a function of the Lewis acidity of the *cus* on the oxide support at a propylene carbonate conversion of 80 %. b) Influence of the addition of increasing amounts of exogenous water on the initial reaction rate (•) and methanol selectivity (▴) for the hydrogenation of propylene carbonate catalyzed by Cu/TaO_*x*_@γ‐Al_2_O_3_. Reaction conditions: *P* ${}_{H{}_{2}}$ =40 bar, initial propylene carbonate concentration=0.25 m in 1,4‐dioxane, catalyst concentration=0.15 mm Cu. Lines in b) were added as guide to the eye.

Carbon dioxide may be formed by direct decarboxylation of propylene carbonate [pathway (iii) in Figure 6 a] or by carbonate hydrolysis in the presence of H_2_O [pathway (iv)]. In turn, water may be formed in situ in the reaction medium as the product of secondary intermolecular dehydration reactions (etherification with methanol and/or oligomerization) of the 1,2‐propanediol primary product, as summarized schematically in Figure 6 b. Both routes undesirably contribute to depletion of the glycol product and thus disable its recycling as CO_2_ carrier in an overall process of CO_2_ utilization. These secondary reactions are known to be acid‐catalyzed. Indeed, a detailed analysis of the reaction products arising from the propylene moiety in the carbonate reactant revealed that the extent to which secondary dehydration reactions take place is greater for catalysts supported on oxides exposing *cus* of markedly Lewis acidic nature. As shown in Figure 7 a, the lumped selectivity to dehydration products, including products of methanol cross‐etherification with propane(di)ols and propylene glycol oligomers, increased on increasing *η* beyond 2.48 eV.

In view of these results, a higher water concentration in the reaction medium, and thus a stronger driving force for propylene carbonate hydrolysis, is inferred for catalysts supported on oxides bearing strongly Lewis acidic *cus*. However, the selectivity to 1‐propanol, the product of the direct carbonate decarboxylation route [pathway (iii) in Figure 6 a], also increased remarkably on increasing *η* beyond 2.45 eV and dominated over dehydration side products for the most Lewis acidic catalyst, that is, Cu/TaO_*x*_@Al_2_O_3_.

To assess the effect of water on the conversion of propylene carbonate, separate experiments were conducted on the most Lewis acidic catalyst Cu/TaO_*x*_@Al_2_O_3_ in which different amounts of water were intentionally added at the beginning of the reaction. As shown in Figure 7 b, the addition of increasing amounts of exogenous water resulted, as expected, in a steep decrease in methanol selectivity, from approximately 30 to less than 5 % for H_2_O/carbonate initial molar ratios ≥0.1, in favor of CO_2_ as the product of carbonate hydrolysis. In parallel, a significant increase in the initial carbonate consumption rate by a factor of approximately four was also observed. These results evidence that reaction conditions favoring propylene carbonate hydrolysis lead to notably higher carbonate conversion rates alongside remarkably lower methanol selectivity than those registered under standard reaction conditions, that is, without the addition of exogenous water to the reaction medium. It was therefore deduced that carbonate hydrolysis pathways driven by the presence of endogenous water are not the major reaction pathway underlying the production of CO_2_ under the conditions of propylene carbonate hydrogenation. Rather, our results suggest that the direct carbonate decarboxylation pathway dominates and is by and large responsible for the formation of CO_2_ on Cu nanoparticles supported on the most Lewis acidic oxides. Facilitation of the carbonate decarboxylation pathway on oxides of pronounced Lewis acidic character is in agreement with previous predictions based on quantum‐mechanical calculations.^18^

The fact that the catalytic performance correlates with the Lewis acidity of the *cus* exposed on the oxide surface at the periphery of the Cu nanoparticles suggests strongly the involvement of these Lewis centers in the activation of the carbonate reactant. Highly electron‐withdrawing (acidic) Lewis centers promote cleavage of C−O bonds between the carbonate functional group and the propane backbone of the substrate molecule, and hence its unselective decarboxylation. In contrast, centers with weaker electron‐withdrawing character (more Lewis basic) seem to facilitate activation of the electrophilic carbonate functional group in the reactant molecule, which results in significantly reduced overall activation energies and notably higher conversion rates. However, the enhanced reactivity is in this case largely owing to the promotion of C−O bond cleavage in the carbonate group, leading to undesired decarbonylation of the substrate. Oxides bearing *cus* Lewis centers of intermediate acidity, such as those exposed on ZrO_*x*_, are essential to favor the selective hydrogenation of the carbonate group in the reactant to form methanol. Moreover, mild Lewis acidity of the oxide support effectively suppresses undesired secondary reactions of the glycol side product, such as dehydration and oligomerization, which are catalyzed by stronger Lewis acid sites. Such preservation of the propanediol backbone of the carbonate molecule enables its recycling as CO_2_ carrier in an overall cyclic process of CO_2_ hydrogenation to methanol. These findings underscore the central role of the oxide support at the periphery of Cu nanoparticles in determining the catalytic performance. Moreover, they serve to clearly identify the relative Lewis acidity (electron‐withdrawing character) of coordinatively unsaturated cationic sites on the oxide surface as a central physicochemical parameter to rationalize and optimize such metal–oxide promotion effects, which play a pivotal role in a number of catalytic reactions of environmental significance, such as hydrogenation of CO_2_ to alcohols^19^ and CO^20^ chemical vectors, or the selective conversion of biogenic oxygenates.3b, 3d

## Conclusions

The deposition of monolayer‐content overlays of various transition‐metal and lanthanide oxides on a high‐surface‐area γ‐Al_2_O_3_ carrier resulted in oxide support materials with essentially identical porosity, while exposing coordinatively unsaturated metal sites (*cus*) with a wide range of surface Lewis acidity. The dispersion of copper nanoparticles on these oxide supports enabled a systematic assessment of metal–support promotion effects in the selective hydrogenation of propylene carbonate to methanol. Under relevant reaction conditions, the overall (apparent) activation energy for the reaction scales with the relative electron‐withdrawing character of the *cus* on the oxide at the periphery of the Cu nanoparticles, as quantified by UV/Vis spectroscopy with 1,2‐dihydroxyanthraquinone as probe molecule, owing to the involvement of oxide Lewis centers in the activation of the carbonate reactant. Moreover, the selectivity of the reaction also correlates with the Lewis acidity of the *cus* on the oxide support. Lewis sites of lower acidity, that is, lower electron‐withdrawing power, promote low‐activation‐energy, and hence faster albeit unselective, decarbonylation of the carbonate reactant. In contrast, Cu nanoparticles interfaced with oxides bearing strongly Lewis acidic sites favor a decarboxylation route. Oxide supports exposing sites of intermediate Lewis acidity are optimal for hydrogenation of the carbonate functional group and maximize the selectivity to methanol. In addition, mild Lewis acidity of the oxide carrier is also essential to inhibit secondary, acid‐catalyzed reactions of the propanediol primary product, to enable its effective recovery and reuse, as required to realize a cyclic process of hydrogenation of waste CO_2_ to methanol mediated by the condensed carbonate derivative. The insights achieved with this set of model catalysts are significant for the design of optimized copper catalysts for the selective hydrogenation of CO_2_‐derived carbonates to methanol. More broadly, they contribute towards a unifying and quantitative description of copper–oxide promotion effects, which play a crucial role in a large number of selective hydrogenation reactions of significance for sustainable chemistry.

## Experimental Section

### Catalyst synthesis

A high‐surface‐area γ‐Al_2_O_3_ carrier was synthesized from a nanosized pseudo‐boehmite precursor (Disperal‐P2, Sasol Materials). A synthesis gel containing the alumina precursor, the polyethylene glycol ether nonionic surfactant Tergitol 15‐S‐7 (Sigma–Aldrich) as porogen agent, and water, with a molar composition of Al/EO/H_2_O of 1:1.51:43 (EO represents the ethylene oxide building units in the polymer) was hydrothermally treated at 383 K in an oven for 48 h. Then, the gel was dried at 353 K for 48 h, at 393 K for 5 h, and finally air‐calcined in a muffle oven at 873 K (3 K min^−1^) and sieved in the 100–200 μm size range for further catalyst synthesis steps.

A series of oxide support materials was synthesized by deposition of monolayer amounts of different transition‐metal and lanthanide oxides (MO_*x*_; M=Sm, Y, Sc, Zr, Ta) on the γ‐Al_2_O_3_ support. Metal precursors were selected on the basis of previous studies13a, 13b to achieve strong interaction with the γ‐Al_2_O_3_ surface. Sm(NO_3_)_3_ **⋅**6 H_2_O (99.9 %, Sigma–Aldrich), Y(NO_3_)_3_ **⋅**6 H_2_O (99.8 %, Sigma–Aldrich), Sc(NO_3_)_3_ **⋅** *x* H_2_O (99.9 %, Sigma–Aldrich), Zr(OC_3_H_7_)_4_ (70 wt % in 1‐propanol, Sigma–Aldrich), and Ta(OC_2_H_5_)_5_ (99.98 %, Sigma–Aldrich) were used as received. Stock solutions were prepared by dissolving nitrate precursors in Milli‐Q water, Zr(OC_3_H_7_)_4_ in dry 1‐propanol, and Ta(OC_2_H_5_)_5_ in dry ethanol. The γ‐Al_2_O_3_ support was dried at 523 K for 3 h under dynamic vacuum (3 mbar). Then, metal/lanthanide incorporation was achieved by incipient wetness impregnation. In all cases, the metal content was adjusted to achieve a surface‐specific metal content corresponding to the experimentally determined monolayer, that is, 4.5–5.0 M_at_ nm^−2^.13a The as‐impregnated solid was transferred into a quartz packed‐bed reactor, dried at 343 K for 10 h, and calcined at 773 K for 4 h under a flow of synthetic air (heating rates of 3 K min^−1^). The calcined solid was transferred to a glovebox under exclusion of air. The series of thus‐synthesized support materials was denoted MO_*x*_@Al_2_O_3_, in which MO_*x*_ stands for the overlay oxide.

Copper was incorporated on the surface of the series of MO_*x*_@Al_2_O_3_ support oxides by incipient wetness impregnation of Cu(NO_3_)_3_ **⋅**3 H_2_O (99 %, Sigma–Aldrich) dissolved in 0.25 m HNO_3_(aq). The Cu concentration of the stock solution was adjusted to attain preset surface‐specific Cu contents of 1.5, 3.0, or 4.5 Cu_at_ nm^−2^. The as‐impregnated solids were transferred to a quartz packed‐bed reactor, dried at 343 K for 10 h, and calcined at 623 K (3 K min^−1^) for 4 h under N_2_ flow. Then, the catalysts were transferred to a U‐shaped packed‐bed glass reactor and activated by reduction at 523 K (1 K min^−1^) in flow of 10 vol % H_2_/N_2_. Lastly, the reactor was allowed to cool to RT, and the solid was recovered, transferred under exclusion of air to a glovebox (O_2_ <0.1 ppm, H_2_O <0.1 ppm), and ground into a fine powder.

### Characterization methods

Nitrogen physisorption isotherms were recorded with a Micromeritics ASAP instrument (3Flex) unit after degassing the sample (≈100 mg) at 423 K under vacuum for 5 h. Specific surface areas were derived by using the BET method in the relative pressure (*P*/*P*_0_) regime of 0.05–0.30. Total mesopore volumes were obtained from the amount of N_2_ taken up at *P*/*P*_0_=0.95. Pore size distributions and average mesopore diameters were determined by applying the BJH method to the desorption branch of the isotherms.

Powder XRD patterns were collected with a STOE Theta/Theta diffractometer by using graphite‐monochromatized CuK_α_ radiation (*λ*=1.5406 Å) with a step size of 0.02° and a dwell time of 3 s step^−1^.

UV/Vis spectroscopy with alizarin (1,2‐dihydroxyanthraquinone) as surface probe molecule was applied to assess the Lewis acidity of the *cus* exposed on the surface of the oxide supports, as reported previously.^14^ The solids were dried and soaked in a stock solution of the probe in dry ethanol (0.15 mm) with exclusion of air. After solid recovery by filtration, excess (unbound) alizarin was washed off with dry ethanol and the solid was dried at RT under vacuum (3 mbar). Diffuse‐reflectance UV/Vis spectra were recorded with a PerkinElmer Lamda 365 spectrometer by using BaSO_4_ as reflectance standard and converted to absorption by using the Kubelka–Munk formalism. The peak energy for the IMCT band *E*_IMCT_ of the adsorbed probe was determined after subtraction of the spectra for the support material prior to alizarin uptake.

H_2_‐TPR experiments were performed with a Micromeritics Autochem 2910 device. First, the sample was flushed with Ar at 393 K for 2 h (10 K min^−1^). Materials containing SmO_*x*_ and YO_*x*_ overlays, of markedly basic character, were additionally calcined in situ at 773 K (10 K min^−1^) for 4 h under a flow of 5 % O_2_/Ar to decompose surface carbonate species that might have developed through uptake of atmospheric CO_2_ during specimen manipulation and experimental setup. After cooling to RT, the temperature was ramped to 1073 K (5 K min^−1^) under a flow of 10 % H_2_/Ar. Evolved water was removed from the outlet stream by a cold trap (195 K), and the H_2_ consumption profiles were recorded with a thermal conductivity detector (TCD).

HAADF‐STEM and EDX studies were performed with a C_s_‐corrected dedicated STEM microscope (Hitachi HD‐2700) equipped with a cold‐field emission gun and two EDAX Octane T Ultra W EDX detectors and operated at 200 kV. Prior to observation, the reduced catalysts were embedded in a low‐viscosity resin (Spurr, Sigma– Aldrich) in a glovebox. The resin was then cured in an oven at 333 K overnight. Specimen cross sections with a nominal thickness of 50 nm were obtained with a DIATOME diamond knife mounted on a Reichert Ultracut ultramicrotome and collected on a nickel TEM grid (400 mesh) coated with a Lacey carbon film (PLANO).

XP spectra were collected with a customized spectrometer equipped with a hemispherical SPECS PHOIBOS 100 analyzer in fixed‐transmission mode at 20 eV pass energy. Spectra were acquired with a nonmonochromatic dual X‐ray source (MgK or AlK radiation) with an anode current of 20 mA and a potential acceleration of 12 kV. As‐calcined samples were pressed into small disks and evacuated in a pre‐chamber at 423 K and <10^−7^ mbar. Then, catalyst reduction was performed in a high‐temperature SPECS HPC‐20 reaction cell with IR heating. In this case, the samples were treated in a flow of 20 vol % H_2_/Ar, by heating from RT to 543 K (3 K min^−1^) and holding the final temperature for 2 h at 1 bar. After cooling to RT, the samples were evacuated at <10^−7^ mbar and transferred to the chamber of the spectrometer. Given the low surface carbon content after the in situ reduction treatment, BEs were referred to the Al 2p signal from γ‐Al_2_O_3_ at 74.10 eV. To derive surface relative atomic ratios, peak intensities were determined after nonlinear Shirley‐type background subtraction and corrected by sensitivity factors (Scofield). Average Cu particle sizes were derived from the experimental Cu/Al surface ratios by using the Kerkhof–Moulijn model^21^ for high‐surface‐area supported catalysts, modified to consider a monolayer of the corresponding MO_*x*_ oxide on the surface of the Al_2_O_3_ carrier.

### Catalytic experiments

Catalytic experiments were performed in a polytetrafluoroethylene (PTFE)‐lined 36 mL autoclave reactor. The pre‐reduced catalyst powder (<20 μm) was loaded into the reactor under exclusion of air in a glovebox, in which the Ar overpressure was kept constant at 3 mbar. In a typical experiment, anhydrous propylene carbonate (0.210 g, 2 mmol, 99.7 %, Sigma–Aldrich), anhydrous 1,4‐dioxane as solvent (8 mL, 99.8 %, Alfa‐Aesar), and an amount of catalyst corresponding to 75 μmol of Cu were added into the PTFE liner of the autoclave. The reactor was then sealed, taken out of the glovebox, and dosed with H_2_ (Air Liquide, 99.999 %) up to a total pressure of 40 bar at RT. All catalytic tests were performed with vigorous magnetic stirring (1000 rpm). The temperature in the reactor was increased to the preset reaction temperature by using an aluminum heating jacket coupled to a heating plate (8 K min^−1^). After selected reaction times, the reaction was quenched by immersing the autoclave in an ice bath. The gas phase was then recovered in a gas bag and analyzed with an Agilent 7890B GC equipped with two sampling loops, the first of which was connected to a Restek RTX‐1 capillary column (60 m) and a flame ionization detector (FID), and the second to two consecutive packed‐bed columns (Agilent HS‐Q 80/120, 1 and 3 m, respectively), a 13X molecular sieve column, and two TCD detectors for the analysis of permanent gases. Argon was used as internal standard for quantification of gaseous products. Liquid‐phase samples were collected and analyzed with an Agilent 6890 GC equipped with a DB‐WAXetr capillary column (15 m) and a TCD detector by using 1,3‐propanediol and 2pentanol as standards. Initial reaction rates were determined by linear regression of analyses taken at reaction times in the range from 0 to 60 min. Product selectivities are reported on a molar basis at preset propylene carbonate conversions, and independently for products arising from either the O−C−O (methanol, dimethylether, and carbon oxides) or the propane‐backbone (C_3_ alcohols, oligomers thereof, and hydrocarbons) “synthons” in the propylene carbonate reactant.

## Conflict of interest

*The authors declare no conflict of interest*.

## Supporting information

As a service to our authors and readers, this journal provides supporting information supplied by the authors. Such materials are peer reviewed and may be re‐organized for online delivery, but are not copy‐edited or typeset. Technical support issues arising from supporting information (other than missing files) should be addressed to the authors.

Supplementary
