# Supplementary material for: Recycling of CO2 by Hydrogenation of Carbonate Derivatives to Methanol: Tuning Copper–Oxide Promotion Effects in Supported Catalysts
Source: ChemSusChem. 2020 Mar 13;13(8):2043–52. doi: 10.1002/cssc.202000166 (PMC7216934; doi:10.1002/cssc.202000166)
Supplement: Supplementary file 1 — Supplementary [file CSSC-13-2043-s001.pdf]

ChemSusChem

Supporting Information

Recycling of CO_2 by Hydrogenation of Carbonate Derivatives to Methanol: Tuning Copper–Oxide Promotion Effects in Supported Catalysts

Jonglack Kim,[a] Norbert Pfänder,[b] and Gonzalo Prieto*[a]

TABLE OF CONTENTS:

Supplementary figures and schemes

Scheme S1	2
Figure S1	3
Figure S2	4
Figure S3	5
Figure S4	6
Figure S5	7
Figure S6	8
Figure S7	9
Figure S8	10
Figure S9	11

Supplementary Tables

Table S1	12
Table S2	13

References 14

Supplementary figures

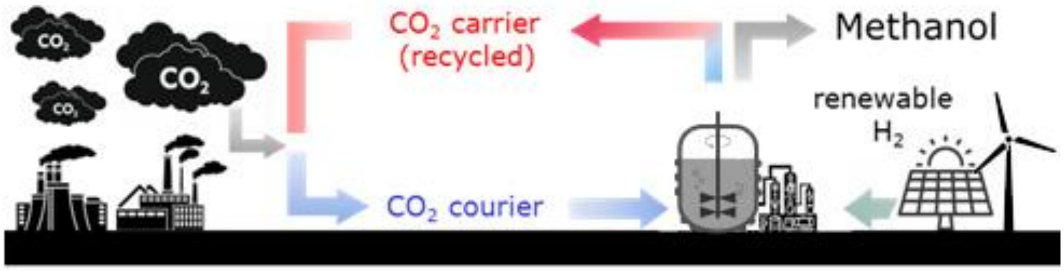

Scheme S1: Conceptual representation of the indirect hydrogenation of CO_2 to methanol via a condensed organic derivative, e.g. an organic carbonate, carbamate or urea, as intermediate *CO_2 courier* compound. The organic intermediate serves to bridge remote point sources of waste CO_2 and renewable H_2, as well as to buffer intrinsic fluctuations of both sources. The term "*CO_2 carrier*" denotes the organic reagent, e.g. alcohol, glycol, amine, etc which is first used to fixate CO_2 into a condensed compound and then recycled to realize an overall cyclic process of hydrogenative CO_2 recycling into methanol.

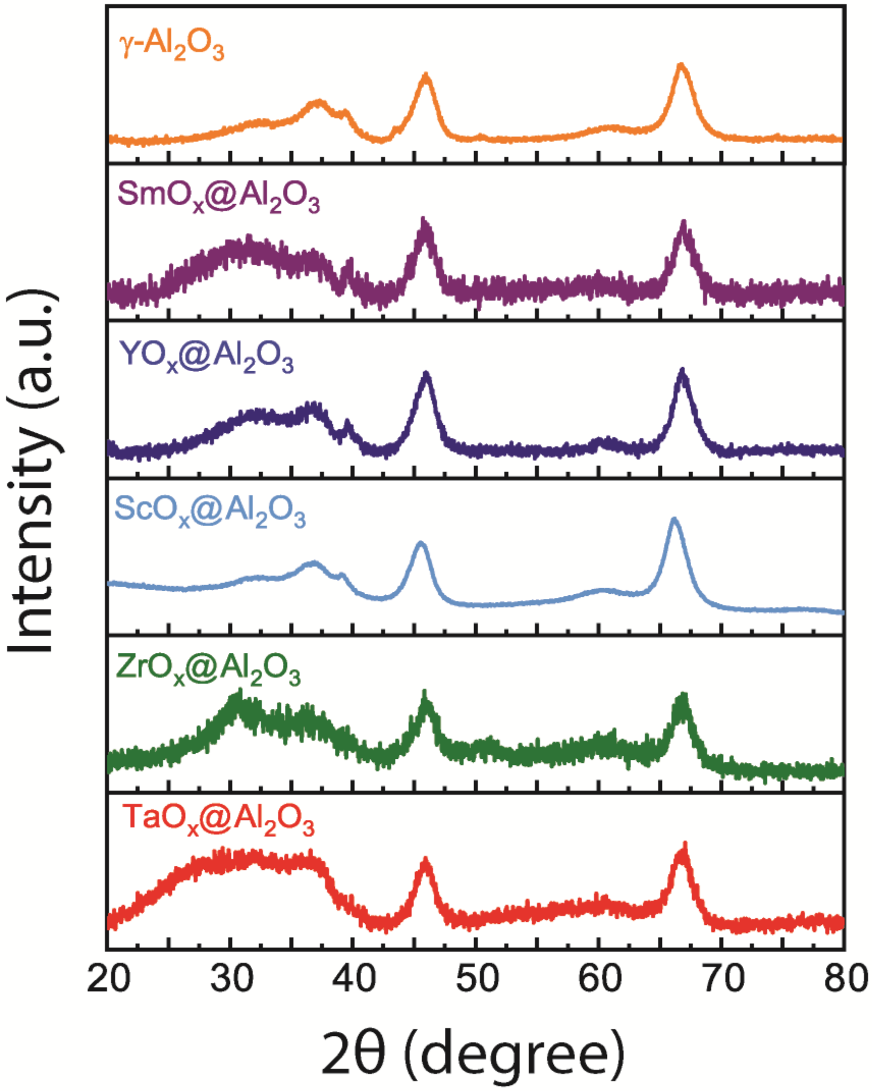

Figure S1: X-ray diffractograms for the series of $MO_x@Al_2O_3$ support materials. The difractograms have been recorded with a CuKα radiation (λ= 1.5406 Å).

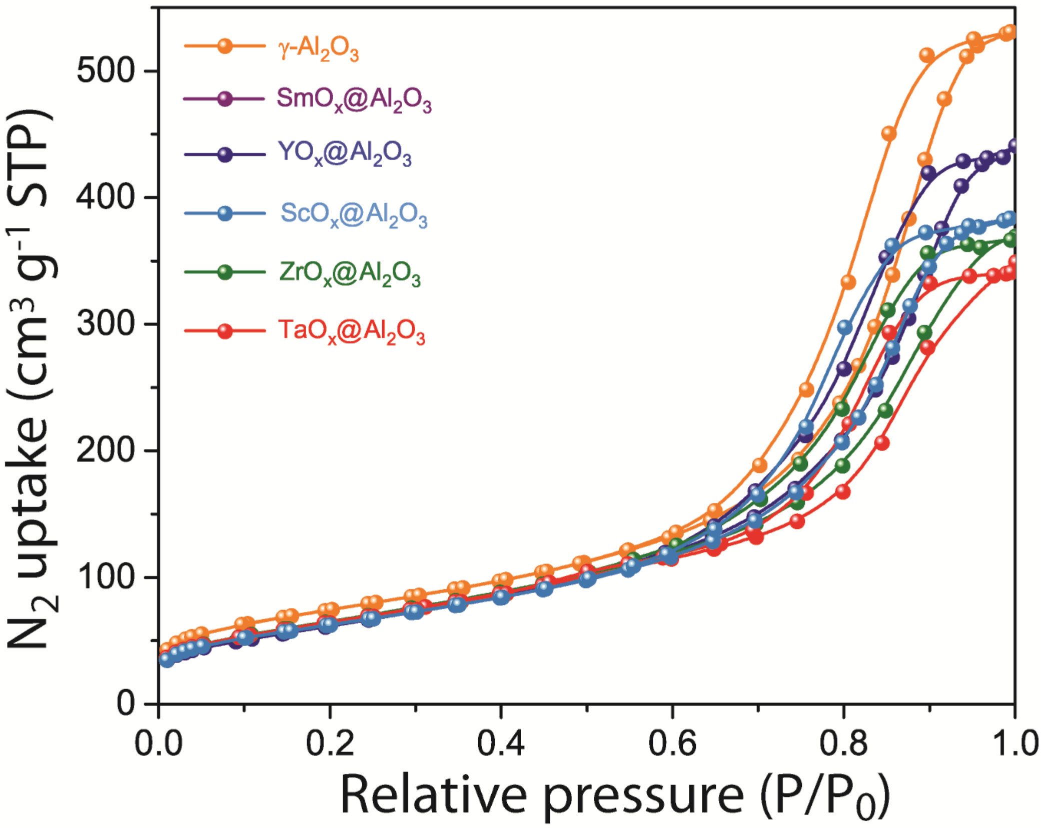

Figure S2: N_2 physisorption isotherms for the series of MO_x@Al_2O_3 support materials..

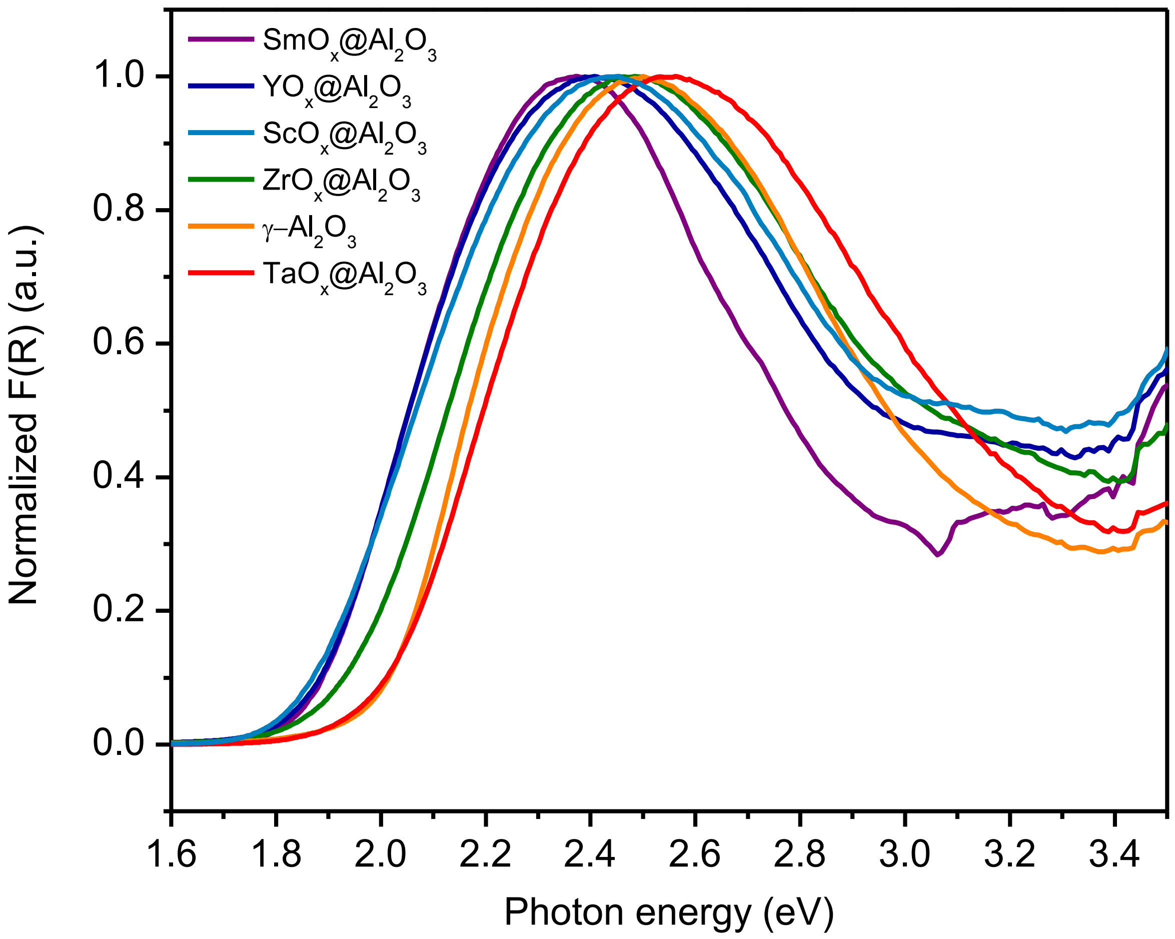

Figure S3: UV-Vis spectra for the series of $MO_x@Al_2O_3$ support oxides, after saturation absorption of 1,2-dihydroxyanthraquinone, in the spectral region for the intramolecular charge-transfer (E_{IMCT}) of the adsorbed probe molecule.

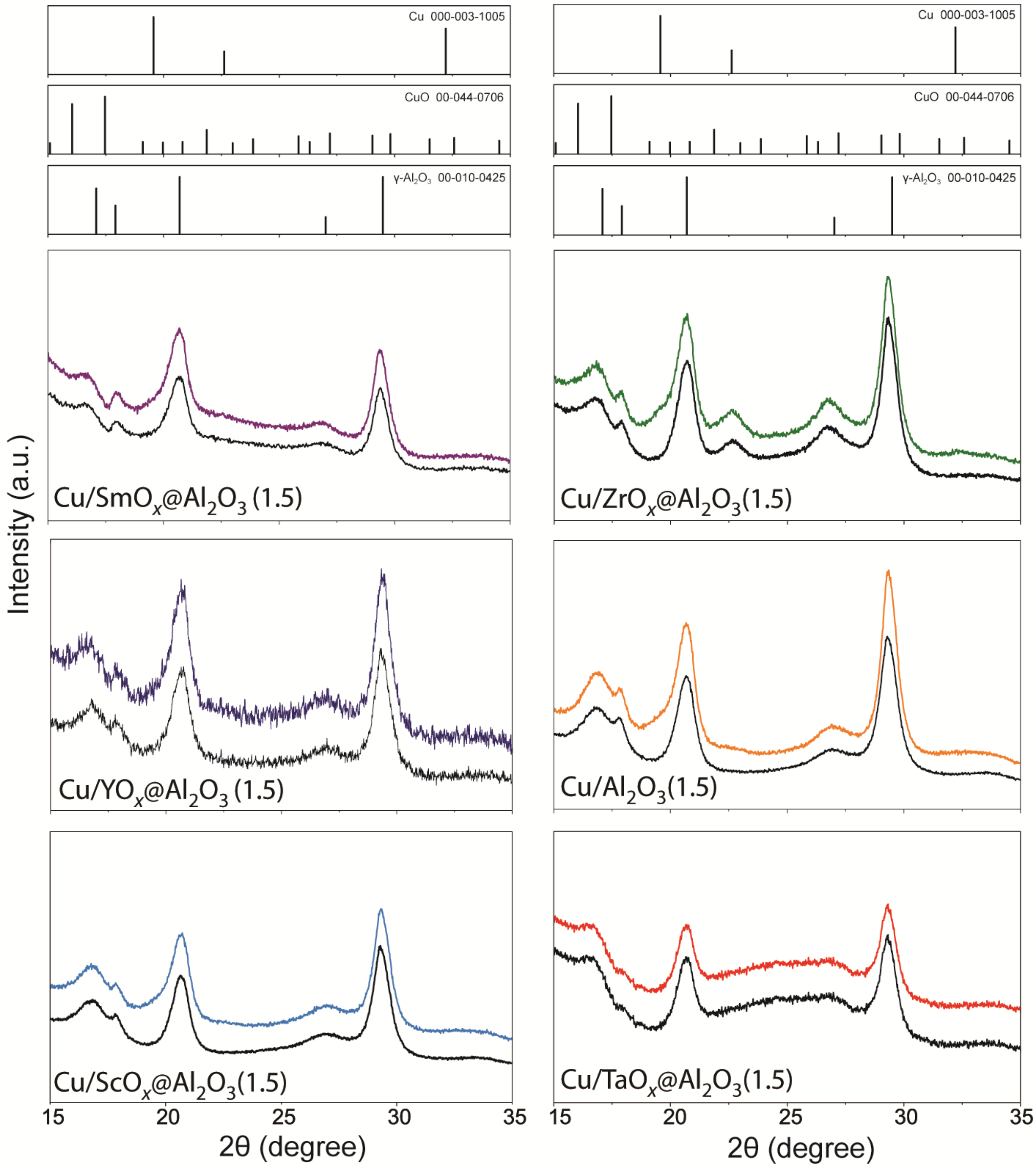

Figure S4: X-ray diffraction patterns for Cu/MO_x@Al_2O_3 catalysts with a Cu content of 1.5 Cu_{at} nm^{-2} in their as-synthesized (bottom, black traces) and as-reduced (top, color traces) states. The diffractograms have been recorded with a MoKα radiation (λ= 0.7093 Å). The diffraction patterns for CuO, γ-Al_2O_3 and metallic Cu have been included for reference.

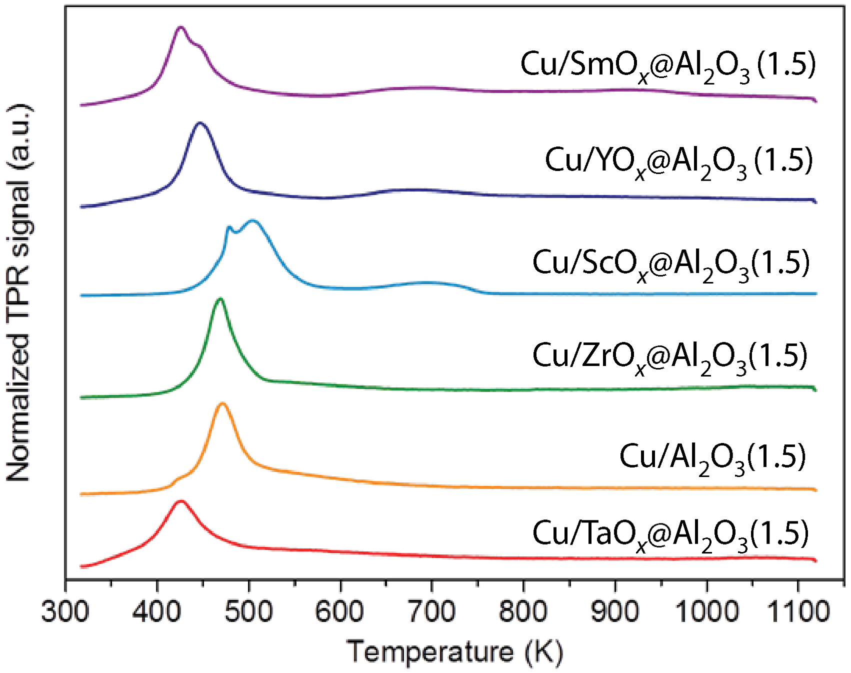

Figure S5: H_2-temperature programmed reduction (H_2-TPR) profiles for the series of Cu/MO$_x$@Al$_2$O$_3$ catalysts (M=Sm, Y, Sc, Zr, Al and Ta). The signal has been normalized to the amount of Cu.

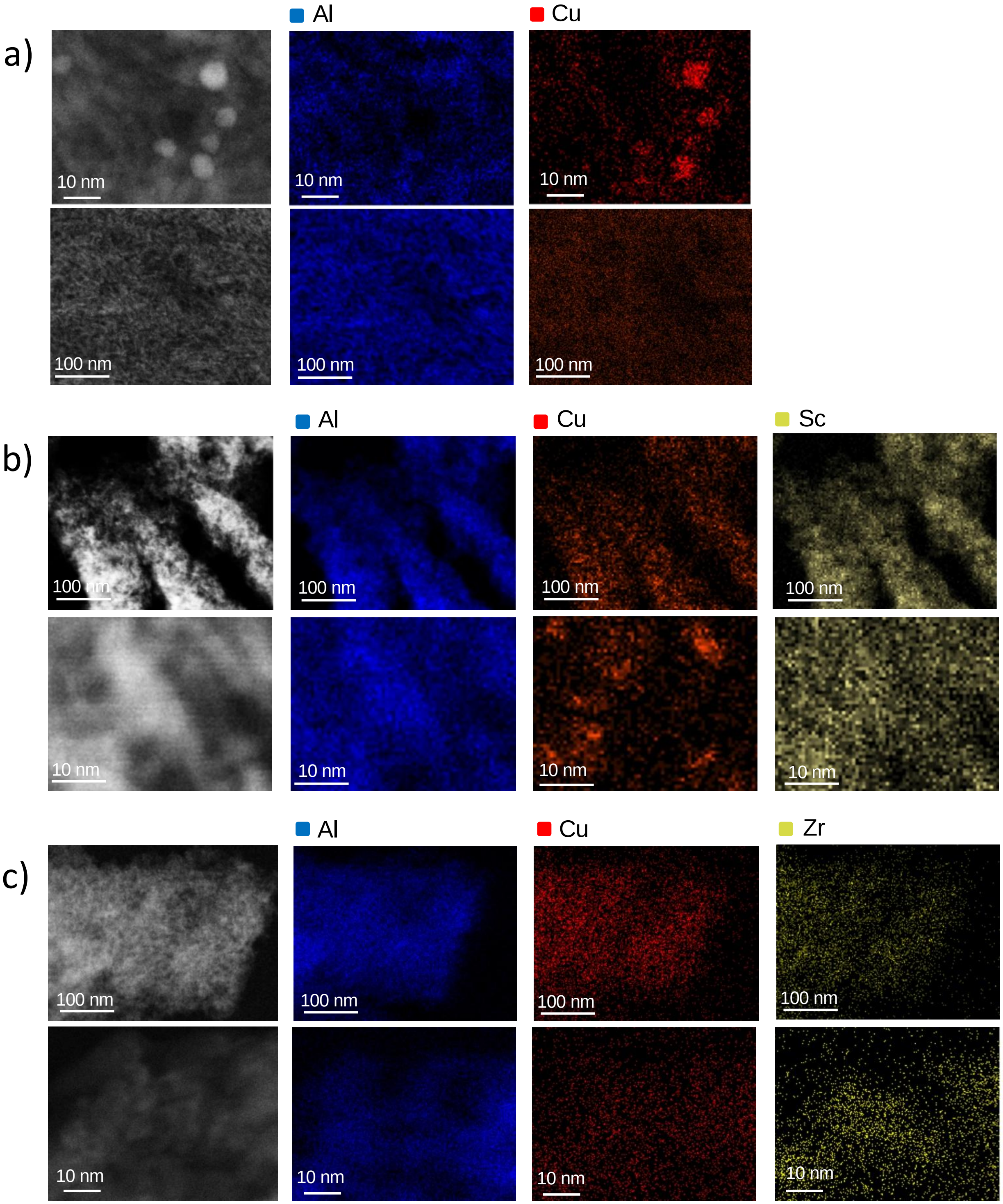

Figure S6: High-Angle Annular Dark-Field Scanning Electron (HAADF-STEM) micrographs (left panel) and the corresponding Energy Dispersive Spectroscopy (EDX) compositional maps for a) Cu/Al_2O_3, b) $Cu/ScO_x@Al_2O_3$ and c) $Cu/ZrO_x@Al_2O_3$ after reduction. The copper content is in all cases 1.5 Cu_{at} nm^{-2}.

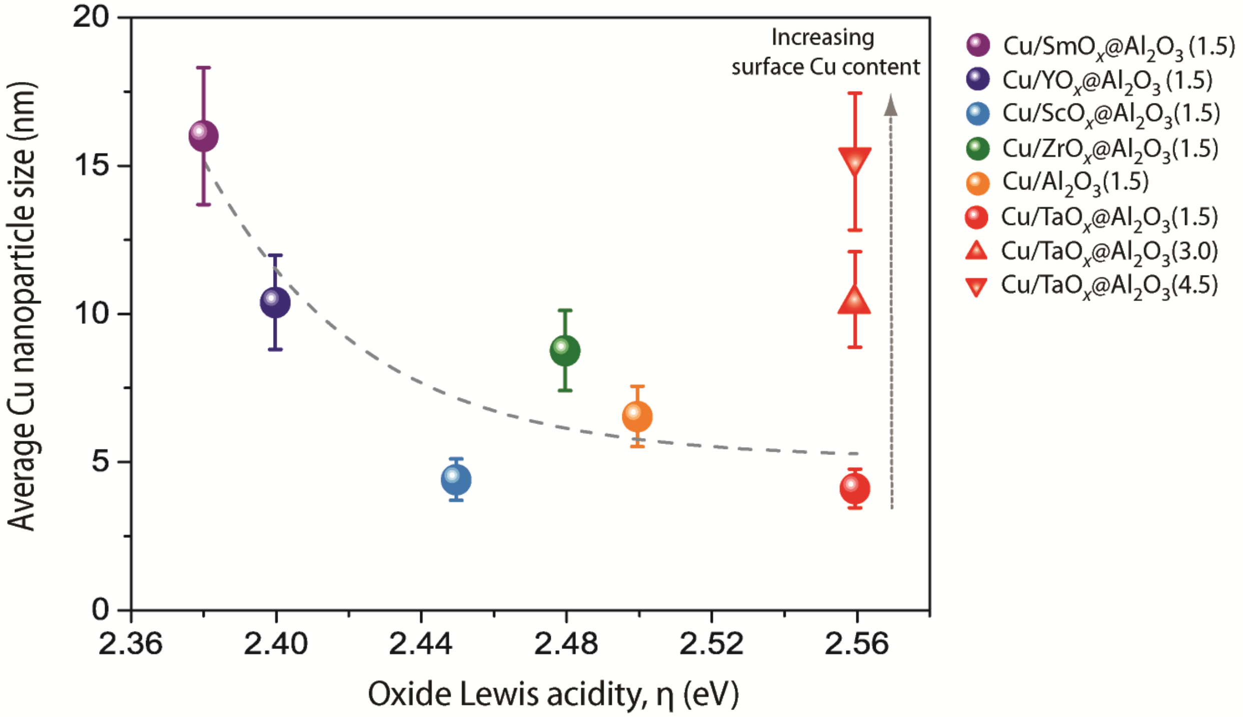

Figure S7: Average Cu nanoparticle size, as determined by a quantitative analysis of the XPS spectra for the *in situ* reduced $Cu/MO_x@Al_2O_3$ catalysts according to the Kerkhof-Moulijn model,[1] as a function of the Lewis acidity of the oxide support as represented by the spectroscopic parameter η. Values in brackets in the catalyst name on the legend give the Cu content (Cu_{at} nm^{-2}).

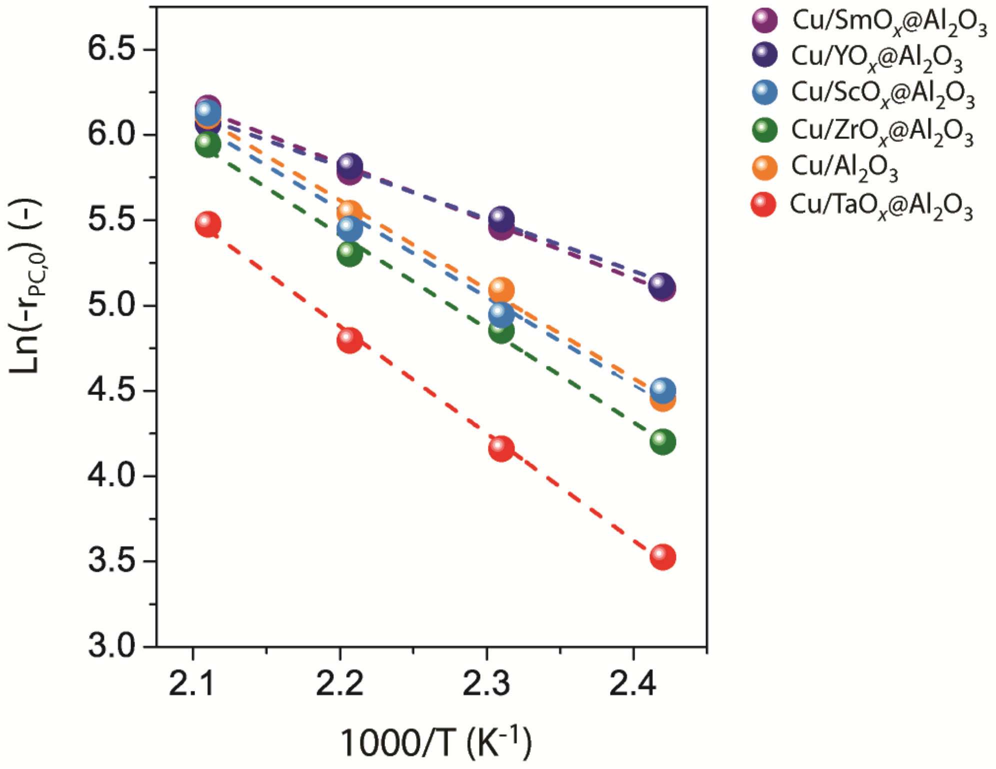

Figure S8: Arrhenius plots in the range of 413-473 K for the hydrogenation of propylene carbonate with different Cu/MO$_x$@Al$_2$O$_3$ catalysts with a Cu content of 1.5 Cu$_{at}$ nm^{-2}. Reaction conditions: P_{H2} =40 bar, initial propylene carbonate concentration= 0.25 M in 1,4-dioxane, catalyst concentration adjusted in each case to achieve a propylene carbonate conversion ≤30% in 60 minutes).

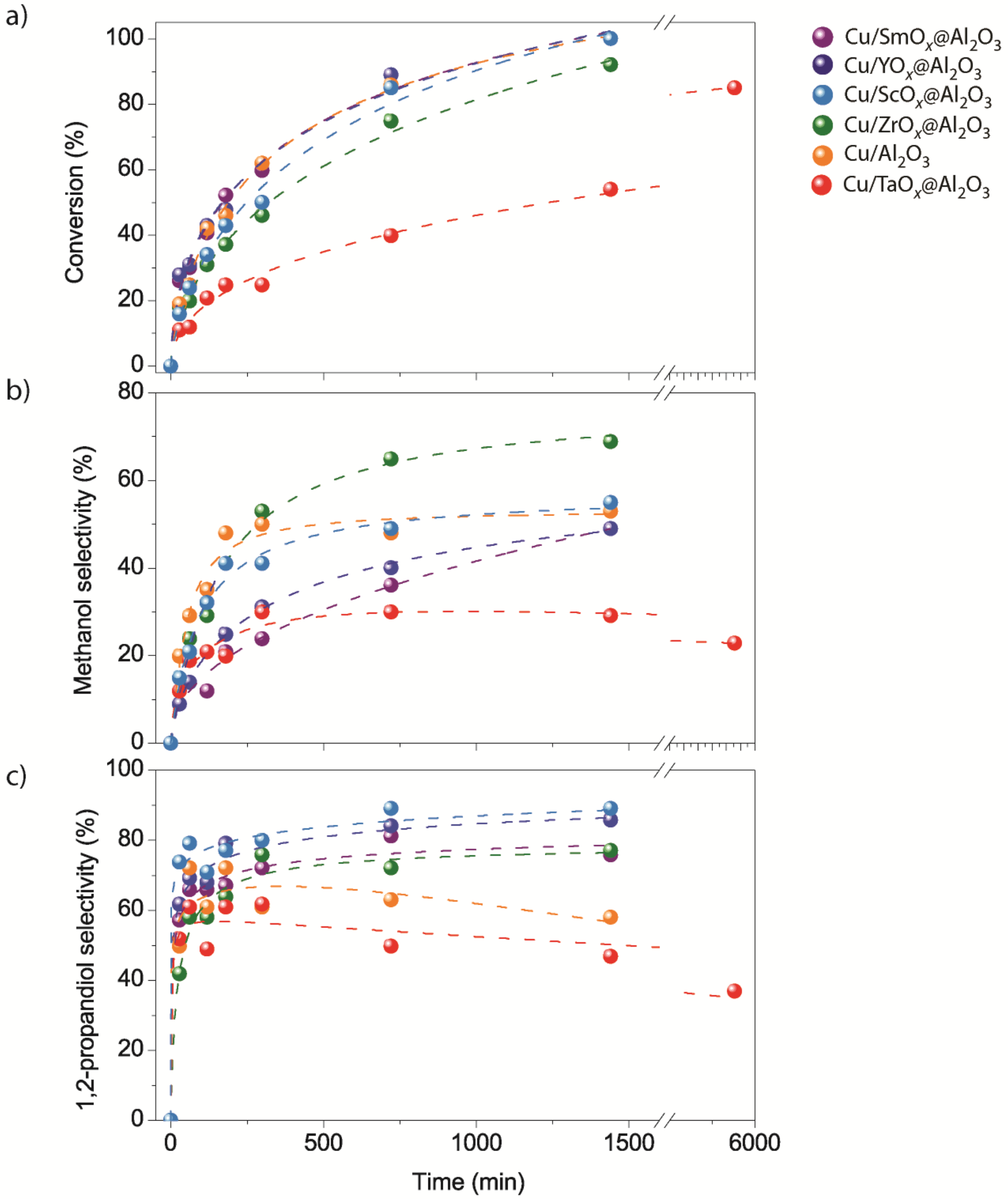

Figure S9: Time resolved evolution of (a) the conversion of propylene carbonate, b) the selectivity to methanol, and c) the selectivity to 1,2-propanediol in the hydrogenation of propylene carbonate catalyzed by the series of Cu/MO$_x$@γ-Al$_2$O$_3$ catalysts (M= Sm, Y, Zr, Al and Ta, Cu content 1.5 Cu$_{at}$ nm^{-2}). Reaction conditions: T= 453 K, P_{H2} =40 bar, initial propylene carbonate concentration= 0.25M in 1,4-dioxane, catalyst concentration = 0.15 mM Cu. The legend in the plot denotes the identity of the oxide support.

Supplementary Tables

Table S1: XPS results for the series of $Cu/MO_x@Al_2O_3$ catalysts after reduction.

Catalyst[a]	$Cu2p_{3/2}$ B.E. (eV)	M[b] B.E. (eV)	M[b] B.E. Assignment
$Cu/SmO_x@Al_2O_3$ (1.5)	932.3	$Sm3d_{5/2}$ 1090.2	Sm(III)
$Cu/YO_x@Al_2O_3$ (1.5)	932.5	$Y3d_{5/2}$ 158.0	Y(III)
$Cu/ScO_x@Al_2O_3$ (1.5)	931.9	$Sc2p_{3/2}$ 402.1	Sc(III)
$Cu/ZrO_x@Al_2O_3$ (1.5)	931.7	$Zr3d_{5/2}$ 182.0	Zr(IV)
Cu/Al_2O_3 (1.5)	932.7	Al2p 74.1[c]	Al(III)
$Cu/TaO_x@ Al_2O_3$ (1.5)	932.3	Ta4f7/2 22.2<br>Ta4f7/2 26.0	Ta(*X*) 0<*X*<5<br>Ta(V)
$Cu/TaO_x@ Al_2O_3$ (4.5)	931.4[d]	$Ta4f_{7/2}$ 22.3<br>$Ta4f_{7/2}$ 26.0	Ta(*X*) 0<*X*<5<br>Ta(V)

[a] In brackets the Cu content (Cu_{at} nm^{-2})
[b] M: transition metal or lanthanide element in the overlay MO_x oxide.
[c] Applied as calibration reference for binding energies.
[d] Cu $L_3M_{4,5}M_{4,5}$ Auger signal at K.E.=919 eV.

Table S2: Initial methanol formation rates determined in the hydrogenation of propylene carbonate or CO_2 with selected $Cu/MO_x@Al_2O_3$ catalysts.

Catalysts	(r_{MeOH}) [mmol g_{Cu}^{-1} min^{-1} L^{-1}]		$[(r_{MeOH})_{CO2}/(r_{MeOH})_{PC}]_{max}$ [c]
	PC[a]	CO_2[b]	
$Cu/SmO_x@Al_2O_3$	7	29	0.2
$Cu/ZrO_x@Al_2O_3$	17	40	0.07
$Cu/TaO_x@Al_2O_3$	23	21	0.02

[a] Reaction conditions: $[PC]_0$= 0.25 M, T= 453 K, P_{H2} =40 bar (at room temperature), solvent: 1,4-dioxane, catalyst concentration: 0.15 mM Cu. PC= propylene carbonate.

[b] Reaction conditions: P_{CO2}10 bar (at room temperature), P_{H2} =40 bar (at room temperature), T= 453 K, solvent: 1,4-dioxane, catalyst concentration: 0.15 mM Cu.

[c] Maximum ratio of methanol formation rates from propylene carbonate (primary methanol production pahway) and CO_2 (secondary methanol production pathway) under those reaction conditions applied herein for the hydrogenation of propylene carbonate. This upper limit for the ratio of methanol formation rates from each is estimated from the pseudo-first order (see discussion below) kinetic rate constants determined experimentally for each of the carbon substrates, and considering the case where the CO_2 partial pressure in the reactor corresponds to that expected if all the propylene carbonate converted at a conversion level of 25% had been equimolarly transformed to CO_2 via direct decarboxylation (<0.5 mmol CO_2).

According to kinetic model proposed by Graaf et al.[2] the formation rate of methanol via hydrogenation of CO_2 can be expressed as:

$$r_{CH3OH(CO2)} = k_{CH3OH} b_{CO2} \left\{ \frac{P_{CO2} P_{H2}^{3/2} - \dfrac{P_{CH3OH} P_{H2O}}{P_{H2}^{3/2} K_{CH3OH(CO2)}}}{(1 + b_{CO} P_{CO} + b_{CO2} P_{CO2}) \left[P_{H2}^{3/2} + \left(\dfrac{b_{H2O}}{b_{H2}^{1/2}} \right) P_{H2O} \right]} \right\} \qquad (16)$$

where k_{CH3OH} is the rate constant, b_x denotes the adsorption equilibrium constant for compound *x*, P_x represents the partial pressure of compound *x*, and $K_{CH3OH(CO2)}$ is the equilibrium constant for the reaction of hydrogenation of CO_2 to methanol. Assuming a very low magnitude for the adsorption equilibrium constants b_x [2, 3] and a low CO_2 conversion (leading to negligible water and methanol partial pressures), Eq. 16 can be reduced to Eq. 17, i.e. methanol synthesis displays a pseudo-first order kinetics with respect to CO_2.

$$\boldsymbol{r_{CH3OH(CO2)}} \cong k_{CH3OH} b_{CO2} P_{CO2} \qquad (17)$$

References

1. F. P. J. M. Kerkhof and J. A. Moulijn, *The Journal of Physical Chemistry*, 1979, **83**, 1612-1619.

2. G. H. Graaf, E. J. Stamhuis and A. A. C. M. Beenackers, *Chem. Eng. Sci.*, 1988, **43**, 3185-3195.

3. G. H. Graaf, H. Scholtens, E. J. Stamhuis and A. A. C. M. Beenackers, *Chemical Engineering Science*, 1990, **45**, 773-783.
